# Supplementary material for: Chromothripsis during telomere crisis is independent of NHEJ, and consistent with a replicative origin
Source: Genome Res. 2019 May;29(5):737–49. doi: 10.1101/gr.240705.118 (PMC6499312; doi:10.1101/gr.240705.118)
Supplement: Supplemental Material [file supp_gr.240705.118_Supplemental_file_1.zip › contigs/annotated_contigs/DB56/contig.2.DB56_length_627_mean_cov_4.42105263158.docx]

**DB56_length_627_mean_cov_4.42105263158**

```
ATCTGGTGCTCTAGGGCTAGGATAGAGAGCAGGCAGAGAGAATGTCAGTCGAAGAAGTTAGAACACAGAAAATCAATGTATCCTCACAT
 >chr4:152182382-152182782 - E=5e-229
TGGAATATTCCAGTATAGCCCCATCCAGTCACCCAGCAGGAGGTTGTATTCTTCTTCAGGTAGACTTCAGATGAGGGAAGGCAGATGAG

CAGGACAACAGGGCTAACAGAAACTGGGGAGGGCAGTTCTACCACAGCGATGGCACTAGATGTGTTTGCTTGGAAATCAGGGTGGGGGG

ATAATCTGGGACACAGGTATTATCGTCATTTTGGAGTCTGCACAACCAAAGATCTGAGCCCACCAATACACTGTATTTCCTGGCATCCT

TAGACCTGAGAAGAGAAGCAGAAGAGATACCGTGGGTTTCT|TGT|ATAGTTAAAGGTGATGAGGAATCAATGATAAAGCTGGAAACAG
                                          >chr4:152703891-152704121 - E=1e-126
GGGCTGGGACAATGGTTCTCCAAGCATGTGTGAGTTGCTTTTAGGGCCTTAAGTAAAGATGCCCAAGGAAATGAGGCATCGGGGAGCAA

TGTAAATTCATGATCTGAAGATTCAGTACAGCTGGCCCCTGATGCTCTGTGGAAAAAAGGGGATGAAATGTTGCTCAATCTGTTTTCCA

AGGAGA
```
